# Subgroup analysis of continuous renal replacement therapy in severely burned patients

**DOI:** 10.1371/journal.pone.0189057

**Published:** 2017-11-30

**Authors:** Jaechul Yoon, Youngmin Kim, Dohern Kym, Jun Hur, Haejun Yim, Yong-Suk Cho, Wook Chun

**Affiliations:** Department of Surgery and Critical Care, Burn Center, Hangang Sacred Heart Hospital, College of Medicine, Hallym University Medical Center, Youngdeungpo-gu, Seoul, Korea; University of Sao Paulo Medical School, BRAZIL

## Abstract

Continuous renal replacement therapy (CRRT) is administered to critically ill patients with renal injuries as renal replacement or renal support. We aimed to identify predictors of mortality among burn patients receiving CRRT, and to investigate clinical differences according to acute kidney injury (AKI) status. This retrospective observational study evaluated 216 Korean burn patients who received CRRT at a burn intensive care unit. Patients were categorized by AKI status. Data were collected regarding arterial pH, laboratory results, ratio of arterial oxygen partial pressure to fractional inspired oxygen (PF ratio), and urine production. Among surviving patients, CRRT duration and the sequential organ failure assessment score were 6.5 days and 4.7 in the non-AKI group and 23.4 days and 7.4 in the AKI group, respectively (p = 0.003 and p = 0.008). On logistic regression analyses, mortality was significantly associated with a pH of <7.2 (p = 0.004), potassium levels of >5.0 mEg/L (p = 0.045), creatinine levels of >2.0 mg/dL (p = 0.011), lactate levels of >2 mmol/L (p<0.001), a PF ratio of <200 (p = 0.042), and a platelet count of <100,000/μL (p<0.001). In the AKI group, poor outcomes were associated with a pH of <7.2, potassium levels of <5.0 mEg/L, lactate levels of >2 mmol/L, and a platelet count of <100,000/μL, while good outcomes were associated with creatinine levels of >2 mg/dL. In the non-AKI group, poor outcomes were associated with lactate levels of >1.5 mmol/L, a PF ratio of <200, and a platelet count of <100,000/μL, while good outcomes were associated with creatinine levels of >1.2 mg/dL. Duration of the CRRT application and the requirement for either renal replacement or renal support at the initiation of CRRT application are important considerations depending on its application.

## Introduction

Burn injuries have devastating physical, physiological, and psychological effects, and are associated with high rates of morbidity and mortality. The leading causes of death among major burn cases are sepsis (78–85% of all burn-related deaths) [1, 2] and multiple organ dysfunction syndrome [3]. Of multiple organ dysfunctions, acute kidney injury (AKI) is a common complication in burn patients with an incidence of 1–40% and the associated mortality rate is 50–100% [4]. Although critical care improvements during the last decade have helped decrease the mortality rate among burn cases, burns remain an important global cause of disability and death [5]. Continuous renal replacement therapy (CRRT) is an important critical care treatment that is generally provided to critically ill patients for renal replacement (in cases of AKI) or for renal support (in cases without AKI) [6]. Although the therapeutic mechanisms of CRRT for critically ill patients without acute renal failure are unclear, CRRT is known to remove inflammatory mediators and cytokines in cases of systematic inflammatory response syndrome and sepsis [7], improve oxygenation in patients with acute respiratory distress syndrome (ARDS) [8], and remove fluid without creating a cardiac burden in patients with congestive heart failure [9]. Similarly, some burn patients may need renal replacement, inflammatory mediator or cytokine removal, and volume overload control. Therefore, CRRT is an important treatment for severely burned patients, although there are insufficient studies regarding the efficacy and indications for CRRT in this setting. The present study aimed to retrospectively identify factors that predicted mortality among burn cases treated using CRRT, and to investigate any clinical differences according to whether the patients had AKI (i.e., renal replacement vs. renal support).

## Methods

### Patient selection

This retrospective observational study evaluated data from 216 Korean adult burn cases that were treated using CRRT at a burn intensive care unit between January 2009 and December 2015. The study’s retrospective protocol was approved by the institutional review board of Hangang Sacred Heart Hospital, and the requirement for informed consent was waived. All patient data were anonymized before the analysis.

Patients with chronic renal failure were excluded. All data were retrieved from a prospectively collected clinical database at the Hangang Sacred Heart Hospital (Hallym University, Seoul, Korea). We collected baseline data regarding age, sex, weight, extent of burns, presence of inhalation injury, start of CRRT, and duration of CRRT. The Abbreviated Burn Severity Index (ABSI) scores the severity of burn injuries, and is calculated by adding the numerical scores for age, sex, extent of burns, presence of full-thickness burns, and presence of inhalation injury [10]. The Acute Physiologic and Chronic Health Evaluation Score III (APACHE III) and Simplified Acute Physiology Score (SAPS) were calculated to assess case severity at the admission and start of CRRT, and the sequential organ failure assessment (SOFA) score was calculated to assess organ failure severity at the start of CRRT. We also identified patients who received vasopressors or supportive care using a ventilator. Furthermore, we collected data from the start of CRRT regarding mean arterial pressure (MAP), arterial pH, laboratory results (potassium, blood urea nitrogen [BUN], lactate, white blood cells [WBC], platelets, total bilirubin [TB], bicarbonate, and creatinine), ratio of arterial oxygen partial pressure to fractional inspired oxygen (PF ratio), and urine production (urine output for 24 h before the start of CRRT divided by body weight and time).

The patients were categorized according to whether they did or did not have AKI, which was diagnosed using the AKIN criteria (Stage 1: creatinine increased 1.5× baseline value or absolute increased creatinine of at least 0.3 mg/dL within 48 hours or urine production of <0.5 mL/kg for 6 h, Stage 2: creatinine increased 2× baseline value or urine production of <0.5 mL/kg for 12 h, Stage 3: creatinine increased 3× baseline value or >4 mg/dL, absolute increased creatinine of at least within 48 h, urine output of <0.3 mL/kg for 24 h, or requiring CRRT regardless of the status. As all patients in this study required CRRT, criteria of “requiring CRRT” in stage 3 were excluded. Data regarding baseline creatinine levels were only included if they were measured within 3 months before the burn injury. If measured results were not available, we estimated the baseline creatinine levels using the modification of diet in a renal disease equation [11]. The CRRT was mainly provided for renal support in the non-AKI group and for renal replacement in the AKI group.

### Intervention

The indications for CRRT at our burn center were acidosis (pH of <7.2), azotemia (BUN of >40 mg/dL), oliguria (urine output of <0.5 mL/kg/h), hyperkalemia (potassium levels of >5.5 mEq/L), volume overload (pulmonary edema/effusion observed during chest radiography), hypercreatinemia (creatinine levels of >2 mg/dL), hyperlactatemia (lactate levels of >2 mmol/L), and the discretion of the attending burn surgeon. CRRT was performed using the continuous veno-venous hemodiafiltration mode, with a blood flow rate of 125 mL/min and an intensity of 2,000 mL/h, regardless of the individual patient’s weight. Hemosol BO^®^ fluid (Gambro) was used as the dialysate and replacement fluid.

### Statistical analysis

Continuous variables were expressed as mean ± standard deviation, and categorical variables were expressed as number and percentage. Normally distributed continuous variables with variance homogeneity were analyzed using the independent *t*-test, and non-normally distributed continuous variables with/-out variance heterogeneity were analyzed using the Mann-Whitney U-test. Categorical variables were analyzed using the chi-square test. The area under the curve (AUC) of the receiver operating characteristic (ROC) curve was used to evaluate the predictive accuracy of mortality for laboratory results. Independent predictors of mortality were categorized with normal reference or cutoff values, which were calculated using the ROC curve with the Youden index or based on equality of sensitivity and specificity. Logistic regression analysis was also performed to evaluate independent associations between the clinical factors and mortality. Results are given as odds ratio (OR) and 95% confidence intervals (CI 95%). Differences were considered statistically significant at p-values of <0.05. All analyses were performed using IBM SPSS software (version 24.0; IBM Corp., Armonk, NY).

## Results

### Characteristics of the survivors and non-survivors, and according to AKI stage

The study identified 216 eligible patients, and we noted 176 deaths (81.5%). The mean age was 52.8 years, and 86.1% of the patients were male. The mean burned total body surface area was 55.8%, and the value was greater among non-survivors (60.4%) than survivors (35.3%). The mean ABSI score was 10.6, with non-survivors having a higher score (11.1) than survivors (8.5). Compared with survivors, non-survivors had significantly more severe acidosis (7.20 vs. 7.26; p <0.001), hyperlactatemia (2.7 mmol/L vs. 1.7 mmol/L; p = 0.002), lower platelet count (95,500/μL vs. 168,200/μL p <0.001), a lower PF ratio (150.7 vs. 197.8 p <0.001), and lower serum creatinine levels (2.0 mg/dL vs. 2.9 mg/dL, p = 0.002) (Table 1).

**Table 1:** Patients’ characteristics and prognostic factors of the survivors and non-survivors, and according to AKIN stage.

	Total (n = 216)	Survivors (n = 40)	Non-survivors (n = 176)	p-value	AKIN0 (n = 26)	AKIN1 (n = 15)	AKIN2 (n = 26)	AKIN3 (n = 149)	p-value
Age (years)	52.8±15.0	54.3±14.2	52.5±15.2	0.550	53.0±17.3	59.3±14.8	55.8±17.8	51.6±14.0	0.221
Sex (male:female)	186:30	36:4	150:26	0.593	24:2	11:4	20:6	131:18	0.163
TBSA burned (%)	55.8±25.2	35.3±23.4	60.4±23.3	<0.001	66.3±24.3	60.5±25.9	45.3±25.4	55.3±24.7	0.016
Inhalation Injury	103(47.7%)	16(40.0%)	87(49.4%)	0.367	13(50.0%)	8(53.3%)	9(34.6%)	73(49.0%)	0.546
Weight	68.2±11.3	68.7±8.9	68.1±11.7	0.438	67.2±13.8	67.5±9.5	65.0±10.7	69.0±11.0	0.320
ABSI score	10.6±2.5	8.5±2.5	11.1±2.3	<0.001	11.8±2.2	11.3±2.7	9.7±2.5	10.5±2.5	0.009
APACHE of admission	52.4±18.9	47.2±18.6	53.6±18.8	0.117	53.6±13.2	45.9±18.9	58.6±15.2	51.8±20.1	0.074
SAPS of admission	42.7±9.9	38.6±9.8	43.6±9.7	0.003	42.6±8.4	40.7±12.3	45.1±10.1	42.5±9.9	0.975
LOSICU (days)	28.9±28.5	58.9±32.7	22.1±22.6	<0.001	30.2±37.5	22.7±12.0	24.4±20.8	30.1±29.2	0.896
LOS (days)	34.3±37.5	87.6±43.4	22.2±22.6	<0.001	36.7±44.8	22.7±12.0	25.9±21.8	36.5±39.8	0.898
CRRT									
Initiation day	12.4±13.3	16.2±12.2	11.6±13.4	0.011	10.6±6.5	13.0±9.4	15.1±16.0	12.2±14.0	0.767
Intensity(mL/kg/h)	30.1±4.7	29.6±4.0	30.2±4.8	0.438	31.0±6.5	30.2±4.1	31.5±5.0	29.6±4.3	0.320
Duration (days)	11.7±13.6	20.8±16.7	9.6±11.9	<0.001	8.0±9.0	9.9±10.2	8.5±9.7	13.1±14.9	0.148
Sepsis	121(56.0%)	22(55.0%)	99(56.2%)	1.000	13(50.0%)	9(60.0%)	11(42.3%)	88(59.1%)	0.388
APACHE III at start	70.7±18.6	60.0±15.6	73.2±18.4	<0.001	56.5±16.0	69.9±18.8	66.6±19.3	74.0±17.7	<0.001
SAPS II at start	49.4±10.8	42.7±9.1	50.9±10.6	<0.001	45.8±9.8	49.0±8.5	48.2±13.2	50.2±10.7	0.055
SOFA at start	8.8±2.9	7.0±2.4	9.2±2.9	<0.001	7.1±3.1	7.9±3.0	8.5±3.0	9.2±2.7	0.003
MAP	59.5±17.0	74.4±19.9	56.0±14.2	<0.001	64.8±18.2	56.9±15.5	60.7±14.5	58.6±17.3	0.403
Supportive cares									
Ventilator	197(91.2%)	26(65.0%)	171(97.2%)	<0.001	24(92.3%)	15(100%)	25(96.2%)	133(89.3%)	0.395
Vasopressors	133(61.6%)	14(35.0%)	119(67.6%)	<0.001	14(53.8%)	10(66.7%)	17(65.4%)	92(61.7%)	0.806
Prognostic Factors									
pH	7.23±0.09	7.26±0.09	7.20±0.08	<0.001	7.24±0.08	7.22±0.07	7.23±0.11	7.20±0.08	0.038
Potassium (mEq/L)	4.7±0.8	4.5±0.7	4.8±0.8	0.104	4.6±0.7	4.6±0.6	4.6±0.9	4.8±0.8	0.386
BUN (mg/dL)	42.7±17.7	41.6±15.2	42.9±18.2	0.890	42.4±18.0	38.6±22.6	38.1±16.5	43.9±17.2	0.264
Creatinine (mg/dL)	2.2±1.4	2.9±2.1	2.0±1.1	0.002	1.1±0.4	1.2±0.5	1.6±0.7	2.6±1.5	<0.001
Urine/kg/h (cc)	0.7±0.6	0.8±0.7	0.7±0.5	0.300	1.5±0.7	1.1±0.4	0.7±0.4	0.5±0.4	<0.001
Lactate (mmol/L)	2.5±1.8	1.7±1.2	2.7±1.9	<0.001	2.1±1.3	2.7±1.7	3.1±2.7	2.5±1.7	0.378
WBC (x10^3^/μL)	17.1±10.2	16.5±11.9	17.2±9.8	0.377	14.8±9.1	18.5±9.9	15.6±9.6	17.6±10.5	0.296
Platelet (x10^3^/μL)	109.0±96.6	168.2±127.8	95.5±82.7	<0.001	131.5±136.6	136.9±117.1	103.9±76.3	103.1±89.0	0.582
PF ratio	159.4±87.2	197.8±63.1	150.7±89.6	<0.001	146.4±75.5	146.0±98.3	161.9±97.9	162.6±86.5	0.725
TB (mg/dL)	1.5±1.4	1.1±0.9	1.6±1.5	0.026	1.1±1.4	1.8±1.4	1.9±1.9	1.5±1.3	0.014
Mortality	176(81.5%)				20(76.9%)	15(100%)	23(88.5%)	118(79.5%)2	0.163

n, number; FB, Flame Burn; SB, Scald Burn; EB, Electrical Burn; ChB, Chemical Burn; CoB, Contact Burn; %TBSA burned, percentage of total body surface area burned; LOSICU, length of stay in ICU stay; LOS, length of hospital stay

We also divided into groups according to AKIN stage. There were significant differences in the percentage of TBSA burned, ABSI score, APACHE III score and SOFA score at CRRT start. In prognostic factors, pH, creatinine, urine per body weight per hour and total bilirubin showed significant differences among AKIN stages. (Table 1)

The order of indications for CRRT was hyperlactatemia, acidosis, azotemia, oliguria, hyperkalemia, volume overload and hyperkalemia. The proportion of oliguria and hypercreatinemia were increased statistical significantly as AKIN stages (p <0.001). (Table 2).

**Table 2:** Indication for CRRT application according to AKIN stage.

	Total (n = 216)	AKIN0 (n = 26)	AKIN1 (n = 15)	AKIN2 (n = 26)	AKIN3 (n = 149)	p-value
Acidosis	110(50.9%)	10(38.5%)	6(40.0%)	13(50.0%)	81(54.4%)	0.385
Azotemia	108(50.0%)	14(53.8%)	5(33.3%)	11(42.3%)	78(52.3%)	0.429
Oliguria	97(44.9%)	1(3.8%)	0(0%)	10(38.5%)	86(57.7%)	<0.001
Hyperkalemia	32(17.8%)	3(11.5%)	1(6.7%)	3(11.5%)	25(16.85%)	0.640
Overload	58(26.9%)	10(38.5%)	7(46.7%)	3(11.5%)	38(25.5%)	0.046
Hypercreatinemia	90(41.7%)	1(3.8%)	1(6.7%)	7(26.9%)	81(54.4%)	<0.001
Hyperlactatemia	117(54.2%)	11(42.3%)	9(60.0%)	17(65.4%)	80(53.7%)	0.390

### Characteristics and prognostic factors between AKI and non-AKI group

We also divided into survivor and non-survivor group according to the presence of AKI. In the non-AKI group (AKIN stage 0), the non-survivors had significantly more severe acidosis (7.21 vs. 7.32, p = 0.043), lower creatinine levels (1.0 mg/dL vs. 1.4 mg/dL, p = 0.021), higher lactate levels (2.4 mmol/L vs. 1.1 mmol/L, p = 0.001), lower platelet counts (92,000/μL vs. 263,000/μL, p = 0.039) and a lower PF ratio (124.7 vs. 218.7, p = 0.021) than the survivors. In the AKI group (AKIN stage 1 to 3), significant survival-related differences were observed for pH (7.25 vs. 7.20, p = 0.003), serum potassium levels (4.5 mEq/L vs. 4.8 mEq/L, p = 0.039), serum creatinine levels (3.2 mg/dL vs 2.2 mg/dL, p = 0.001), serum lactate levels (1.8 mmol/L vs. 2.8 mmol/L, p = 0.001), platelet counts (151,400/μL vs. 96,000/μL, p < 0.001), PF ratio (194.1 vs. 154.0, p = 0.001), and total bilirubin levels (1.2 mg/dL vs. 1.6 mg/dL, p = 0.034) (Table 3).

**Table 3:** Patients’ characteristics and prognostic factors of the non-AKI and AKI group.

	Non-AKI(N = 26)		p-value	AKI (N = 190)		p-value
	Survivor (N = 6)	Non-Survivor (N = 20)		Survivor (n = 34)	Non-Survivor (n = 156)	
Mean age (years)	49.8±16.2	54.0±17.9	0.620	55.1±13.9	52.3±14.9	0.319
Sex (male:female)	6:0	18:2	1.000	30:4	132:24	0.785
Weight	68.0±13.6	67.0±14.3	0.880	68.8±8.1	68.2±11.4	0.400
TBSA burned (%)	46.2±22.5	72.3±21.9	0.022	33.4±23.3	58.9±23.1	<0.001
Inhalation Injury	4(66.7%)	9(45.0%)	0.642	12(35.35)	78(50.0%)	0.172
ABSI score	9.5±1.9	12.6±1.8	0.006	8.3±2.5	11.0±2.2	<0.001
APACHE at admission	54.5±13.5	53.4±13.5	0.856	45.9±19.2	53.6±19.4	0.074
SAPS at admission	42.0±10.9	42.8±7.8	0.852	37.9±9.6	43.7±9.9	0.002
LOSICU (days)	47.3±31.8	25.1±38.2	0.005	60.9±32.8	21.8±19.9	<0.001
LOS (days)	75.0±46.8	25.1±38.2	0.003	89.9±43.1	21.8±20.0	<0.001
CRRT						
Initiation day	13.0±6.2	9.9±6.5	0.151	16.7±12.9	11.8±14.1	0.131
Intensity(mL/kg/h)	30.4±5.9	31.2±6.7	0.800	29.5±3.7	30.0±4.5	0.400
Duration	6.5±4.6	8.4±10.1	0.951	23.4±16.9	9.8±12.1	<0.001
Sepsis	1(16.7%)	12(60.0%)	0.163	21(61.8%)	87(55.8%)	0.654
APACHE III at start	45.5±18.5	59.8±14.0	0.053	62.5±13.8	74.9±18.2	<0.001
SAPS II at start	40.2±9.9	47.5±9.3	0.032	43.2±9.0	51.3±10.7	<0.001
SOFA at start	4.7±2.3	7.8±3.0	0.021	7.4±2.2	9.4±2.8	<0.001
MAP	80.1±23.2	60.2±14.0	0.015	73.8±19.5	55.5±14.1	<0.001
Supportive cares						
Vasopressors	1(16.7%)	13(65.0%)	0.106	13(38.25)	106(67.9%)	0.002
Ventilator	4(66.7%)	20(100.0%)	0.070	22(64.7%)	151(96.85)	<0.001
Prognostic Factors						
pH	7.32±0.10	7.21±0.05	0.043	7.25±0.08	7.20±0.09	0.003
Potassium (mEg/L)	4.7±0.6	4.5±0.7	0.487	4.5±0.7	4.8±0.8	0.039
BUN (mg/dL)	46.7±21.9	41.1±17.0	0.509	40.7±14.0	43.2±18.4	0.617
Creatinine (mg/dL)	1.4±0.5	1.0±0.3	0.021	3.2±2.2	2.2±1.1	0.001
Urine/kg/h (cc)	1.9±0.7	1.4±0.6	0.144	0.6±0.4	0.6±0.4	0.524
Lactate (mmol/L)	1.1±0.5	2.4±1.3	0.001	1.8±1.3	2.8±1.9	0.001
WBC (x10^3^/μL)	10.9±5.6	15.9±9.7	0.239	17.5±12.5	17.4±9.8	0.688
Platelet (x10^3^/μL)	263.0±224.8	92.0±64.3	0.039	151.4±98.6	96.0±85.0	<0.001
PF ratio	218.7±105.4	124.7±49.2	0.021	194.1±54.1	154.0±93.1	0.001
TB (mg/dL)	0.8±0.3	1.3±1.6	0.951	1.2±1.0	1.6±1.5	0.034

n, number; %TBSA burned, percentage of total body surface area burned; LOSICU, length of stay in ICU stay; LOS, length of hospital stay

We also compared non-AKI and AKI groups among survivors. There were statistical differences in the duration of CRRT (6.5 days vs. 23.4 days; p = 0.003), APACHE III score (45.5 vs. 62.5; p = 0.011), and SOFA score (4.7 vs. 7.4; p = 0.008) at the start, creatinine level (1.4 mg/dL vs. 3.2 mg/dL; p = 0.006), and urine output (1.9 cc/kg/h vs, 0.6 cc/kg/h; p = 0.008).

### AUC of ROC curve of the prediction of mortality at the time of CRRT application

AUCs were calculated to evaluate the accuracy of mortality prediction. Overall, the AUC of platelets was highest at 0.730 and the next was lactate at 0.700. In the non-AKI group, the AUCs of pH and lactate were 0.833, while the AUC was 0.829 for creatinine, 0.817 for the PF ratio, and 0.783 for platelets. In the AKI group, the AUC of platelets was 0.715, creatinine was 0.681, lactate was 0.678, the PF ratio was 0.675, and pH was 0.65. The AUCs of the non-AKI group was higher those of the AKI group for the factor mentioned above. (Table 4)

**Table 4:** AUC of ROC curve for the prediction of mortality at the time of CRRT application.

	AUC (95% CI) in Total	AUC (95% CI) in non-AKI	AUC (95% CI) in AKI
pH	0.680(0.588–0.773)	0.833(0.596–1.000)	0.657(0.559–0.756)
Potassium	0.582(0.490–0.674)	0.358(0.111–0.606)	0.613(0.515–0.710)
BUN	0.507(0.416–0.598)	0.375(0.098–0.652	0.527(0.433–0.622)
Creatinine	0.656(0.561–0.750)	0.829(0.587–1.072)	0.681(0.582–0.780)
Urine	0.553(0.454–0.651)	0.675(0.419–0.931)	0.535(0.431–0.639)
Lactate	0.700(0.610–0.790)	0.833(0.671–0.996)	0.678(0.578–0.779)
WBC	0.545(0.447–0.643)	0.658(0.426–0.891)	0.522(0.416–0.628)
Platelet	0.730(0.647–0.813)	0.783(0.499–1.000)	0.715(0.627–0.803)
PF ratio	0.696(0.624–0.767)	0.817(0.504–1.000)	0.675(0.599–0.751)
TB	0.613(0.517–0.709)	0.508(0.272–0.745)	0.616(0.509–0.723)

### Predictors of mortality according to the presence of AKI

To perform logistic regression analysis for the association of different factors with mortality, clinical factors were categorized as follows; pH of <7.2, BUN of >40 mg/dL, urine output of <1.0 mL/kg/h, potassium level of >5.0 mEq/L, creatinine level of >2.0 mg/dL, lactate level of >2 mmol/L, PF ratio of <200, WBC counts of >12,000/μL, platelet counts of <100,000/μL, and total bilirubin of >1.2 mg/dL. Creatinine and lactate levels were categorized differently at >1.2 mg/dL and >1.5 mmol/L in the non-AKI group. Among all patients who received CRRT, the significant factors in the univariate logistic regression analysis were a pH of <7.2 (OR: 2.932, p = 0.004), potassium levels of >5.0 mEg/L (OR: 2.438, p = 0.045), creatinine levels of >2.0 mg/dL (OR: 0.400, p = 0.011), lactate levels of >2 mmol/L (OR: 3.992, p < 0.001), a PF ratio of <200 (OR: 2.089, p = 0.042), and platelet counts of <100,000/μL (OR: 3.980, p <0.001). Furthermore, in the univariate logistic regression analysis for the non-AKI group, creatinine levels of >1.2 mg/dL (OR: 0.035, p = 0.008), lactate levels of >1.5 mmol/L (OR: 11.667, p = 0.040), a PF ratio of <200 (OR: 18.000, p = 0.011), and platelet counts of <100,000/μL (OR: 11.667, p = 0.040) were significant factors. The significant factors in the AKI group were pH of <7.2 (OR: 2.777, p = 0.011), potassium levels of >5.0 mEg/L (OR: 3.248, p = 0.021), creatinine levels of >2.0 mg/dL (OR: 0.351, p = 0.009), lactate levels of >2 mmol/L (OR: 3.256, p = 0.003), and platelet counts of <100,000/μL (OR: 3.425, p = 0.002). The presence of sepsis, which is a known risk factor, was not significantly associated with mortality in this study group. (Table 5).

**Table 5:** Univariate logistic regression for predicting mortality according to presence of AKI.

	Total		Non-AKI	AKI			
	OR (CI 95%)	p-value	OR (CI 95%)		p-value	OR (CI 95%)	p-value
pH	2.932(1.400–6.137)	0.004	4.091(0.402–41.658)		0.234	2.777(1.266–6.091)	0.011
BUN	1.000(0.503–1.987)	1.000	0.164(0.016–1.666)		0.126	1.300(0.616–2.741)	0.491
Urine output	1.556(0.724–3.347)	0.257	1.667(0.155–17.894)		0.673	1.499(0.585–3.845)	0.399
Potassium	2.438(1.018–5.838)	0.045	0.500(0.066–3.770)		0.501	3.248(1.190–8.863)	0.021
Creatinine	0.400(0.198–0.807)	0.011	0.035(0.003–0.149)		0.008	0.351(0.160–0.769)	0.009
Lactate	3.992(1.873–8.510)	<0.001	11.667(1.112–122.381)		0.040	3.256(1.482–7.155)	0.003
PF ratio	2.089(1.026–4.255)	0.042	18.000(1.917–168.991)		0.011	1.576(0.726–3.419)	0.250
WBC	1.076(0.529–2.190)	0.839	2.444(0.361–16.547)		0.360	0.903(0.410–1.992)	0.801
Platelet	3.980(1.931–8.200)	<0.001	11.667(1.112–122.381)		0.040	3.425(1.587–7.390)	0.002
Bilirubin	2.082(0.995–4.357)	0.051	1.250(0.112–13.924)		0.856	2.145(0.979–4.699)	0.056
Sepsis	1.052(0.527–2.098)	0.886	7.500(0.733–76.773)		0.090	0.781(0.365–1.670)	0.523

OR, Odd Ration; CI, confidence interval

## Discussion

CRRT was developed to remove metabolic waste and water from patients with AKI, although it is also used as a life-saving intervention for critically ill patients with hypercatabolism and volume overload[7, 12]. Despite the fact that appropriate CRRT can improve outcomes for critically ill patients, the mortality rate among patients who received CRRT in the present study (81.5%) was noticeably higher than the rates of 35–55% in other studies of critically ill patients who were not burned and received CRRT[13, 14]. However, the mortality rate in the present study is within the range of 73–100% from another study of burned patients with AKI[15]. Thus, it is important to understand the clinical factors that can predict prognosis after CRRT, as patients with severe burns still have a high risk of mortality, despite improvements in intensive care and CRRT.

The present study revealed that mortality was predicted by pH, creatinine level, and lactate and platelet counts. In this context, acidosis, hyperlactatemia, and thrombocytopenia generally reflect the severity of organ dysfunction and are associated with poorer prognoses. The present study revealed that high creatinine levels were associated with a lower risk of mortality. Previous studies have also reported that creatinine levels of >3 mg/dL were associated with a lower risk of mortality[16] and that high serum creatinine levels independently predicted better outcomes[17, 18]. However, it is not clear why high creatinine levels are associated with better outcomes. It is possible that high serum creatinine levels reflect the presence of fewer comorbid conditions that produce creatinine (e.g., liver disease, reduced muscle mass, and aging) [18]. Furthermore, lower serum creatinine levels at the start of CRRT might indicate fluid overload, which is associated with a poorer prognosis [19]. We also found that platelet count was a statistically significant factor in both the AKI and non-AKI group. Thrombocytopenia is associated with higher risks of mortality in sepsis cases[20] and ARDS cases[21], and thrombocytopenia may reflect the severity of the disease if CRRT is performed regardless of AKI; this could explain the importance of thrombocytopenia as a risk factor.

In large multicenter study [22], there were no significant differences in mortality, recovery of kidney function, and the rate of nonrenal organ failure between intensive (mean 35.8 ml/kg/h) and less intensive (mean 22.0 mL/kg/h) renal replacement therapy in ICU patients with AKI. However, because most burn patients are generally highly metabolic and have experienced shock or developed ALI/ARDS at the time of CRRT, some burn centers reported that those in shock were prescribed a high intensity dose with a mean 63 mL/kg/h while those not in shock received a mean of 46 mL/kg/h [23]. Our center performed CRRT with a mean intensity of 30.1 mL/kg/h, which was more consistent with a large multicenter study [22], and we did not evaluate the effect of the higher intensity dose of CRRT in this study.

In the present study, one patient without AKI was included in the hypercreatinemia group and oliguria group (Table 2). A patient included in the hypercreatinemia group because the baseline creatinine which was measured within 3 months before the burn injury was very high at 1.95 mg/dL and 2.3 mg/dL at CRRT initiation and 2.1 mg/dL at diagnosis within 48 hours. A patient included in the oliguria group (<0.5 mL/kg/h over 24 hours) had 0.54 mL/kg/h for 6 hours, 0.62 mL/kg/h for 12 hours, and 0.31 mL/kg/h for 24 hours; as the creatinine level was normal, we did not diagnose AKI. It is also interesting that CRRT durations among survivors were 6.5 days in the non-AKI group and 23.4 days in the AKI group. This may be related to the fact that CRRT clearance of metabolic waste was achieved within a week for the non-AKI group, which was associated with a good prognosis. In addition, CRRT was used for renal replacement in the AKI group and renal function returned to normal in approximately 3 weeks, which is also associated with a good prognosis. Therefore, the duration of the CRRT application was different depending on its purpose. The initiation day of CRRT in survivors was not significantly different; however, it was earlier in the non-AKI group (13.0 days vs. 16.7 days) and the SOFA and APACHE III scores significantly lower in non-AKI group. From these results, we inferred that it may be effective to provide renal support using CRRT before the patient deteriorates enough to require renal replacement with CRRT.

The present study has several limitations. First, we did not have definitive data regarding the indications for CRRT, as it was often based on the discretion of the attending physician, which increases the risk of selection bias. Second, the retrospective single-center design is also associated with known risks of bias. However, many patients with severe burns throughout Korea are transferred to our center, as it is the only university-affiliated burn center and has been designated as “The Emergency Center for Burn Care” by the Korean Ministry of Health, Welfare, and Family Affairs. Thus, it is possible that our findings are representative of all Korean burn cases. Third, we did not evaluate the effect of CRRT on survival. Therefore, we cannot determine the usefulness of CRRT in severely burned patients. However, we believe that CRRT is able to improve the condition of those who are in need of CRRT. We hope to perform further studies of severely burned patients to validate the findings of the present study.

## Conclusion

In conclusion, CRRT is an important treatment for critically ill patients and burn cases, which have a high risk of mortality. The present study’s results indicate that, among burn cases with AKI that received CRRT as renal replacement, poor outcomes were associated with a pH of <7.2, potassium levels of <5.0 mEg/L, lactate levels of >2 mmol/L, and a platelet count of <100,000/μL, while good outcomes were associated with creatinine levels of >2 mg/dL. Among burn cases without AKI that received CRRT for renal support, poor outcomes were associated with lactate levels of >1.5 mmol/L, a PF ratio of <200, and a platelet count of <100,000/μL, while good outcomes were associated with creatinine levels of >1.2 mg/dL. The start time, duration of the CRRT application, and the requirement for either renal replacement or support at CRRT initiation are other important considerations depending on its purpose.

## Supporting information

S1 Dataset Datasets of this study. (XLSX)
